# Antiretroviral Outcomes in South African Prisoners: A Retrospective Cohort Analysis

**DOI:** 10.1371/journal.pone.0033309

**Published:** 2012-03-21

**Authors:** Natasha E. C. G. Davies, Alan S. Karstaedt

**Affiliations:** 1 Division of Infectious Disease, Department of Medicine, Chris Hani Baragwanath Hospital and Wits Reproductive Health and HIV Institute, University of the Witwatersrand, Johannesburg, South Africa; 2 Division of Infectious Diseases, Department of Medicine, Chris Hani Baragwanath Hospital and the University of the Witwatersrand, Johannesburg, South Africa; Asociacion Civil Impacta Salud y Educacion, Peru

## Abstract

**Background and Methods:**

Little is known about antiretroviral therapy (ART) outcomes in prisoners in Africa. We conducted a retrospective review of outcomes of a large cohort of prisoners referred to a public sector, urban HIV clinic. The review included baseline characteristics, sequential CD4 cell counts and viral load results, complications and co-morbidities, mortality and loss to follow-up up to 96 weeks on ART.

**Findings:**

148 inmates (133 male) initiated on ART were included in the study. By week 96 on ART, 73% of all inmates enrolled in the study and 92% of those still accessing care had an undetectable viral load (<400copies/ml). The median CD4 cell count increased from 122 cells/mm^3^ at baseline to 356 cells/mm^3^ by 96 weeks. By study end, 96 (65%) inmates had ever received tuberculosis (TB) therapy with 63 (43%) receiving therapy during the study: 28% had a history of TB prior to ART initiation, 33% were on TB therapy at ART initiation and 22% developed TB whilst on ART. Nine (6%) inmates died, 7 in the second year on ART. Loss to follow-up (LTF) was common: 14 (9%) patients were LTF whilst still incarcerated, 11 (7%) were LTF post-release and 9 (6%) whose movements could not be traced. 16 (11%) inmates had inter-correctional facility transfers and 34 (23%) were released of whom only 23 (68%) returned to the ART clinic for ongoing follow-up.

**Conclusions:**

Inmates responded well to ART, despite a high frequency of TB/HIV co-infection. Attention should be directed towards ensuring eligible prisoners access ART programs promptly and that inter-facility transfers and release procedures facilitate continuity of care. Institutional TB control measures should remain a priority.

## Introduction

South Africa has a high incarceration rate of 413/100 000 population [1]. Risk factors for incarceration echo those of HIV acquisition: being young, Black and male, poverty, dysfunctional family and/or social relationships, lower educational attainment, unemployment and drug/alcohol use (although intravenous drug use is uncommon) [2]–[4]. Over one third of inmates are below 25 years of age [5]. An exceptionally high violent crime rate results in almost 30% of inmates being incarcerated in maximum security facilities, with 47% of prison sentences being ten or more years in duration [5]. On average, South Africa's 240 correctional facilities operate at 137% of intended capacity [5].

HIV is an important health problem in prisons globally [2], [3]. South Africa, with approximately 5.6 million HIV-infected individuals, has the world's largest single national burden of HIV [6]. HIV prevalence in prisons often outstrips that in the general population [2]. In South Africa over 16% of adults are HIV-infected [6], increasing to 24% for Black males aged 25–49 years [7]. HIV prevalence in South African prisons is estimated to be between 20–40% [2], [4], [8]. During a recent national HIV counseling and testing campaign, 53 000 inmates were tested; 19% of whom tested HIV-positive [9].

HIV increases susceptibility to tuberculosis (TB). South Africa records the third highest TB incidence globally [10], notifying 948 cases/100 000 population [11]. 70% of these TB patients are HIV-infected [11]. Internationally, TB prevalence amongst prisoners is significantly higher than in the general population [8], [12]–[16]. Thus, in South Africa, HIV-infected inmates are at particularly high risk for developing active TB. Escalating rates of TB and HIV may explain the 800% increase in mortality recorded in South African prisons between 1995 and 2005 [17].

Antiretroviral therapy (ART) significantly reduces HIV/AIDS-associated morbidity and mortality [18]. In state prisons in the United States, between 1995 and 1999, AIDS-related mortality fell over 75% - seemingly following increased ART availability [19]. In New York City, AIDS-related mortality declined from 41 to 6 deaths/10 000 inmates between 1990 and 1998; rates for other causes of mortality remaining unchanged [20]. Importantly, ART significantly reduces TB incidence [21], [22] and TB-associated mortality [23].

In Connecticut, 59% of prisoners on ART for over 6 months were virologically suppressed (viral load <400 copies/ml) with a mean CD4 cell count increase of 74 cells/mm^3^ [24]. Few studies report HIV-related health outcomes among prisoners on ART in resource-limited countries. In Malawi, after 12 months on ART, 61% of inmates were alive and accessing treatment [25]. In Thailand, 72% of 88 inmates were accessing care after a median 18 months on ART [26].

South Africa boasts the world's largest ART programme with ever-expanding coverage facilitating treatment access for special populations, including inmates [7]. The Department of Correctional Services introduced an HIV Treatment Policy in 2007 [27]. ART access for prisoners has gradually improved since [27]. 8 091 inmates (5% of all inmates) are currently on ART [9]. 9% of facilities now provide on-site ART services but inmates in other facilities continue to encounter barriers to off-site ART access [28]. Considering 360 000 inmates are released back into the community in South Africa each year [8], successful provision of ART for prisoner populations – both during incarceration and following release - is of great public health importance. This descriptive study reports on HIV-related health outcomes of inmates accessing ART at a large public-sector ART clinic in South Africa.

## Methods

### Study site

The study was conducted at the outpatient adult ART clinic at Chris Hani Baragwanath Hospital, a 2 700 bed public-sector university hospital in Soweto, South Africa. The clinic manages over 4 000 patients on ART. Free ART has been provided since the public-sector ART programme began in April 2004. The typical starting regimen at the time of the study was stavudine, lamivudine and efavirenz. Patients are seen weekly or fortnightly during ART preparation and then 2, 4, 8 and 12 weeks after initiation. Once responding well to treatment, review occurs 3 or 4 monthly. Patients with complex co-morbidities, advanced disease or complications are seen more frequently.

### Prison site

Inmates included in this study were incarcerated in a prison which was situated in the hospital catchment area. Prisoners mostly came from nearby communities, fewer were from distant areas. The prison, like most, is overcrowded and understaffed; cells built for 40 prisoners holding up to 100 [29]. The prison houses between 10 000 and 11 000 inmates. The maximum security section, which accounts for approximately 3 300 inmates, receives an average of 25 new admissions per day (Monday to Friday) and moves or transfers out approximately 40 inmates per week (personal communication). Most inmates in this study were from maximum security and many were serving long sentences.

### Study population

All adult (>18 years) inmates enrolled onto ART at the clinic between April 2004 and February 2008 were included in the study. HIV testing was voluntary and often occurred only once inmates became clinically ill. Only ART eligible inmates were referred off-site. ART eligibility, defined by national guidelines, was limited to CD4 count <200 cells/mm^3^ and/or World Health Organization (WHO) stage 4 disease. Inmates with higher CD4 cell counts were excluded as they were not referred to the clinic. The cohort included ART-naïve inmates and ART-experienced inmates transferred in from other ART sites (9 inmates) during the study period. Prison medical staff did not provide any ART-related care and clinic doctors provided no outreach services at the prison. Inmates had access to prison medical staff that either managed problems on-site or referred them to hospital as needed. Inmates' medication was collected from the clinic by the guards who accompanied inmates off-site. Once collected, inmates self-retained their ART drugs, storing them in their cell.

### Procedures

Inmates were identified using the clinic's booking records. A retrospective cohort analysis was performed. Data was collected from ART clinic and laboratory records until inmates had been on ART for 96 weeks (or closest available results) or were no longer in care at the clinic. Prison medical records were unavailable.

Baseline information comprised age, sex, past history of opportunistic infections, WHO stage, details of previous ART exposure, baseline CD4 count and viral load, hepatitis B and C status and syphilis serology, ART initiation date and regimen choice. Indicators for progress on ART included sequential CD4 and viral load results at 24, 48 and 96 weeks (or closest available result), hospitalizations, new opportunistic infections, TB events on ART, drug toxicity, and any drug changes. Viral load <400 copies/ml defined virological suppression. Outcome was assessed by death, loss to follow-up and retention in care following release. Educational attainment, reason for incarceration, sentence duration, risk factors for incarceration and HIV and history of substance use or mental illness were not routinely recorded and hence are not reported.

Inter-facility transfers and releases meant some records were incomplete. When data were unavailable, the inmate was excluded from the relevant data set(s), resulting in a variable denominator. Clinical files for five inmates were not located; laboratory records provided minimum data comprising demographic information and laboratory results.

### Ethics Statement

The study was approved by the Human Research Ethics Committee, University of Witwatersrand, Johannesburg and by the Department of Correctional Services Research Committee, Pretoria. Two files were excluded because inmates declined consent for their records to be included.

## Results

### Baseline Characteristics

133 (90%) inmates were male, with a median age of 32 years (range 21–54 years).

The median baseline CD4 cell count was 122 cells/mm^3^ (range 4–241). 29 (21%) inmates had CD4 cell count <50 cells/mm^3^.

At baseline, 71 of 148 (51%) inmates had WHO stage 3 conditions: 69 had pulmonary TB, 2 had chronic diarrhea. WHO stage 4 conditions at baseline comprised 14 patients with extra-pulmonary TB, 3 with Kaposi's sarcoma and one with non-Hodgkin's lymphoma. One patient had Hodgkin's lymphoma. 66 (46%) inmates displayed prior hepatitis B exposure of whom 13 (9%) were surface antigen positive – suggesting active disease. One patient was hepatitis C PCR-positive. All inmates tested RPR-negative for syphilis.

### Virological & Immunological Outcomes (Table 1)

**Table 1:** Virological and immunological outcomes at 24, 48 and 96 weeks.

Week	Median CD4 cell count/mm^3^ (IQR)	% Inmates still on ART* with viral load <400 copies/ml	% Total cohort† with viral load <400 copies/ml
Baseline	122 (50–156)		
24	196 (134–269)	91	75
48	246 (179–330)	91	78
96	356 (254–441)	92	73

* Number of inmates virologically suppressed (111, 115, 108)/number of available viral load results at 24, 48 and 96 weeks or closest available result (122, 126, 118 respectively).

† Number of inmates virologically suppressed at 24, 48 and 96 weeks/all 148 inmates including those who died, defaulted, failed treatment or were transferred out/lost to follow-up.

Abbreviation: IQR: inter-quartile range.

108 (92%) of 118 inmates retained on treatment through 96 weeks (or closest available result) were virologically suppressed (viral load <400 copies/ml), equating to 73% of the 148 inmates initially included in the study. Seven patients switched to second-line drugs due to virological failure (national guideline definition: viral load >5 000 copies/ml on at least 2 occasions). Inmates' CD4 cell counts steadily increased from 122 cells/mm^3^ at baseline to 356 cells/mm^3^ at 96 weeks on ART. After 2 years on treatment, 21 (18%) inmates had attained normal CD4 counts, over 500 cells/mm^3^.

### Clinical Outcomes

#### Hospitalization

37 (26%) patients had a total of 45 admissions, of which 13 (29%) occurred within the first 6 months on ART. TB was the most common reason for admission (17/45, 38%). One patient, who was admitted twice, had both TB and *mycobacterium avium* complex. Other reasons for hospitalization included: other infections (lower respiratory tract infection (LRTI) (5 admissions), chronic active hepatitis B, gastroenteritis, meningitis, meningitis plus LRTI); surgery (human papilloma virus wart resection (2), bilateral breast reduction for gynecomastia, decortication for TB empyema, fistulotomy, incision and drainage of inguinal abscess, esophageal dilatation for achalasia, small bowel obstruction following prior abdominal TB) and other medical conditions (deep vein thrombosis (3), symptomatic hyperlactatemia (2), acute psychosis, diabetes mellitus, drug overdose, non-Hodgkin's lymphoma, obstructive jaundice).

#### Complications

Drug toxicity was common. Overall 75 (53%) inmates experienced 100 different side-effects of variable severity including: painful peripheral neuropathy (32 patients), symptomatic hyperlactatemia (22), lipodystrophy (21), gynecomastia (13) and hyperlipidemia (12). The ART regimen was changed in 38 (23%) of 143 inmates. The most common reason for drug switch was stavudine-associated drug toxicity as this was being prescribed at 40 mg twice daily for inmates weighing over 60 kg (as per national guidelines at the time). Twelve (8%) of 143 inmates were diagnosed with depression, although none severe enough to warrant admission.

#### Tuberculosis (Table 2)

**Table 2:** TB rates pre-ART, at ART initiation and on ART.

TB Event	%	N/Denominator
Total giving history of TB disease at baseline	61	86/141
*Previous TB therapy*	*28*	*42/141*
*On TB therapy at ART initiation*	*33*	*44/133*
Total inmates with new TB episode on ART*	21	31/148
Number of confirmed MDR TB cases	10	6/63

* 4 of 31 inmates had 2 episodes of TB once on ART = 35 new episodes of TB.

Abbreviations: TB: Tuberculosis; ART: antiretroviral therapy; MDR: multidrug resistant.

Out of 133 inmates for whom it was recorded, 44 (33%) were on TB treatment at ART initiation. 86 (61%) of 141 patients gave a history of prior and/or current TB. TB was common before ART initiation; at ART initiation and once established on ART. New TB events once on ART occurred as follows: four in the first 12 weeks, four in weeks 13–24, seven in weeks 25–48, seven in weeks 49–72 and nine in weeks 73–96. In four cases timing was unclear. Pulmonary and extra-pulmonary disease (15 and 16 cases respectively) was equally represented amongst new episodes. Four remaining cases were treated empirically or the site was not recorded. By study end, 96 (65%) inmates had ever received one or more courses of TB treatment. Of eight inmates initiating ART within one month after starting TB treatment, five developed immune reconstitution inflammatory syndrome (IRIS). In total, six (10%) inmates treated during the study had confirmed multidrug resistant TB of whom four had a prior history of TB.

#### Mortality

By study end, nine (6%) patients had died - two deaths occurred in the first four months; all others occurred a year or more after ART initiation. The cause of death (severe sepsis) was known for one patient who died in hospital. The cause was not recorded for eight deaths occurring in prison.

#### Loss to Follow-up

34 (23%) inmates were lost to follow-up: 14 (9%) while still incarcerated; 11 post-release and nine (6%) whose movements could not be traced. Inmates lost to follow-up had a lower median baseline CD4 count of 106 cells/mm^3^ (range 7–217). During the study period, 50 (34%) inmates were moved: 16 (11%) had inter-correctional facility transfers and 34 (23%) were released, of whom 23 (68%) returned to the HIV clinic, with four arranging transfers to other ART sites. It is unknown whether the 11 (32%) patients lost to follow-up post-release accessed ART elsewhere.

## Discussion

Inmates in this study demonstrated very good clinical, immunological and virological outcomes following ART initiation, despite high levels of treated TB. 92% of inmates remaining on treatment through 96 weeks were virologically suppressed. Considering the low average baseline CD4 cell count, mortality and non-TB associated morbidity rates were relatively low. TB was the most common co-morbidity: 65% of inmates had experienced one or more episodes of TB by study end. Inter-facility transfers and inmate release into the community were common reasons for loss to follow-up.

Virological suppression in this prison population was equivalent to that reported in South African, community-based, public-sector ART studies: one study reported 90.8% virological suppression at six months on ART [30], another reported 69.7% virological suppression after 24 months on ART [31]. Inmate populations in developed-world settings display less impressive outcomes with 46–59% virological suppression at six months on ART [24], [32]. However, in developed nations, intravenous drug use amongst HIV-infected inmates frequently impacts treatment adherence and rates of significant co-morbidities [2], [33]. In South Africa, although alcohol and substance misuse is prevalent, intravenous drug use remains uncommon, perhaps partially explaining the noticeably better virological outcomes in this cohort [2], [8]. Directly Observed Therapy (DOTS) for ART has previously been evaluated, with conflicting results [34]–[36]. The success of a non-DOTS or patient-retained approach [33] in this cohort suggests DOTS would yield limited, if any, benefit and may even, as seen elsewhere [37], impact acceptability and uptake of ART services.

The high rate of virological suppression attained in this cohort has significant public health implications. Recently, the HPTN 052 study convincingly showed that sustained virological suppression, following ART initiation, resulted in a 96% reduction in sexual HIV transmission in sero-discordant heterosexual couples [38]. Considering high levels of sexual violence, tattooing practices and violent disputes within prisons, attaining virological suppression in inmates on ART potentially represents an important means of reducing HIV transmission within prisons.

At baseline, inmates had advanced HIV with low median CD4 cell counts, as commonly seen in both prison and community settings. In Thailand, 81% of inmates initiating ART had WHO stage 3 or 4 disease [26]. One large community-based South African study reported a median baseline CD4 count of 103 cells/mm^3^ at ART initiation, with 27% having CD4 counts below 50 cells/mm^3^ [39]. Another South African study reported a median baseline CD4 count of 125 cells/mm^3^ at ART initiation [40]. Initiating ART in those with advanced HIV is associated with higher rates of ART-associated complications, TB and death [41], [42]. Considering mortality may be up to 50% higher in those initiating ART with advanced disease [41], the low early mortality in this group is striking [30], [31]. The relatively high hospitalization rate may have been due to prevalent advanced disease combined with ready hospital access via the ART clinic. The median CD4 cell counts on ART compared favorably with other South African cohorts (288 cells/mm^3^ at two years on ART) [31] and other inmate cohorts (74 cells/mm^3^ at six months) [24]. The frequency of drug toxicities likely arose due to use of twice daily stavudine at 40 mg. Tenofovir is now available in the public-sector so NRTI-associated toxicities occur less commonly. However, considering almost half of inmates had been exposed to hepatitis B, hepatitis B screening will prove an important step before discontinuing tenofovir in those requiring switch to second-line agents due to virological failure [43].

One third of inmates experienced inter-facility transfers or release into the community. Loss to follow-up commonly occurred at these times. Considering inmates' encouraging ART outcomes whilst in prison, any breakdown in continuity of care during inmate movements represents a considerable risk to the sustainability of such positive outcomes [24], [44]. Currently no standardized approach exists to ensure inmates initiating ART in prison successfully connect with community-based, public-sector ART sites post-release. Inmates presenting to community-based clinics without a standard transfer letter may encounter difficulties accessing routine follow-up and consistent ART provision post-release [45]. Disrupted care during inter-facility transfers and post-release has been identified as problematic in other prison-based studies [24], [32], [46], [47]. Such treatment disruptions, and subsequent loss of virological suppression, negatively impact individual patients and public health [33]. Policies should be developed and implemented to address this. Protocols facilitating continuity of care during inmate movement minimize treatment interruptions, ensuring better virological outcomes [46], [47]. Such ‘linkage to care’ or case management interventions have proven highly effective elsewhere [48]–[50]. One study reported that 90% of HIV-infected inmates continued accessing community-based HIV care post-release following the implementation of discharge planning and case management [16]. A recent literature review identified four additional elements of transitional care that significantly impact on treatment outcomes for HIV-infected prisoners post-release: continuity of ART provision; concurrent management of substance use disorders; continuity of management for any mental health conditions and interventions to enhance secondary prevention via reduction in HIV-associated risk-taking behaviors [49].

TB disease was common before ART initiation, at initiation and once established on ART. In comparison, a South African community-based cohort reported TB rates of 14% prior to ART initiation, 5% at initiation and 8% after initiation [42]. Worldwide, TB in prisons exceeds that in the general population [12]–[15]. The increased vulnerability to TB seen in HIV-infected individuals may explain why this cohort of HIV-infected inmates demonstrated markedly higher TB rates than reported in studies of general inmate populations [8], [12]–[16]. The high proportion of inmates on TB treatment at ART initiation may have been because TB diagnosis triggered their referral to the clinic or because pre-ART screening identified inmates with undiagnosed TB. The retrospective nature of the study precludes definite conclusions about whether TB developing once on ART was already present but undiagnosed at ART initiation. However, TB rates throughout the study remained constant and were not higher in the first 3–6 months, suggesting that TB diagnosis pre-ART was not frequently missed. Further research, involving a larger inmate cohort initiating ART, might clarify these points and validate the findings regarding TB frequency. As reported elsewhere, ART-associated TB IRIS was most common when ART was initiated within one month of TB treatment commencing [51]. The MDR rate in this cohort was slightly higher than rates seen in the community but the low numbers mean this finding would need corroboration [52]. The frequency of TB in this study indicates a need for comprehensive implementation of the WHO's ‘3 I's’ approach to TB control (intensified case finding, isoniazid prophylactic therapy and infection control) within correctional facilities [53] and timely ART initiation in TB/HIV co-infected inmates with CD4 cell counts <350 cells/mm^3^ (as per updated South African National guidelines) [43].

The study has limitations usually associated with retrospective reviews. Additionally, the study took place soon after Department of Correctional Services committed to providing HIV care for inmates and so HIV testing uptake was low and off-site referral encountered many barriers. This explains the small cohort size despite the combination of high HIV prevalence and a large prison population. Referral bias may mean this cohort is not fully representative of all ART-eligible inmates. Inmates who accessed HIV testing and secured off-site referral may represent a particularly motivated group of individuals, possibly resulting in excellent treatment adherence. The low mortality seen in this group may also be due to very ill inmates being deemed too sick for off-site care. Consequently, results may not be reproducible if ART care is provided on-site, within a prison facility, or at a less sophisticated hospital.

In conclusion, prisoners do well on ART if referred for treatment in a timely manner. Despite the small cohort size, this study sets an important benchmark for prison ART programmes in resource-limited settings. Considering the individual and public health benefits of effective ART, attention to HIV testing, prompt ART initiation, TB control, and linkage to care during transfers and post-release should be prioritized by prison programmes.
